# Exposure-Response Relationships for Isavuconazole in Patients with Invasive Aspergillosis and Other Filamentous Fungi

**DOI:** 10.1128/AAC.01034-17

**Published:** 2017-11-22

**Authors:** Amit V. Desai, Laura L. Kovanda, William W. Hope, David Andes, Johan W. Mouton, Donna L. Kowalski, Robert W. Townsend, Salim Mujais, Peter L. Bonate

**Affiliations:** a Astellas Pharma Global Development, Inc., Northbrook, Illinois, USA; b University of Liverpool, Liverpool, United Kingdom; c University of Wisconsin, Madison, Wisconsin, USA; d Erasmus MC, Rotterdam, The Netherlands

**Keywords:** isavuconazole, SECURE clinical trial, exposure-response, triazole

## Abstract

Isavuconazole, the active moiety of the water-soluble prodrug isavuconazonium sulfate, is a triazole antifungal agent for the treatment of invasive fungal infections. The purpose of this analysis was to characterize the isavuconazole exposure-response relationship for measures of efficacy and safety in patients with invasive aspergillosis and infections by other filamentous fungi from the SECURE clinical trial. Two hundred thirty-one patients who received the clinical dosing regimen and had exposure parameters were included in the analysis. The primary drug exposure parameters included were predicted trough steady-state plasma concentrations, predicted trough concentrations after 7 and 14 days of drug administration, and area under the curve estimated at steady state (AUCss). The exposure parameters were analyzed against efficacy endpoints that included all-cause mortality through day 42 in the intent-to-treat (ITT) and modified ITT populations, data review committee (DRC)-adjudicated overall response at end of treatment (EOT), and DRC-adjudicated clinical response at EOT. The safety endpoints analyzed were elevated or abnormal alanine aminotransferase, increased aspartate aminotransferase, and a combination of the two. The endpoints were analyzed using logistic regression models. No statistically significant relationship (*P* > 0.05) was found between isavuconazole exposure and either efficacy or safety endpoints. The lack of association between exposure and efficacy indicates that the isavuconazole exposures achieved by clinical dosing were appropriate for treating the infecting organisms in the SECURE study and that increases in alanine or aspartate aminotransferase were not related to increase in exposures. Without a clear relationship, there is no current clinical evidence for recommending routine therapeutic drug monitoring for isavuconazole.

## INTRODUCTION

**FIG 1:**
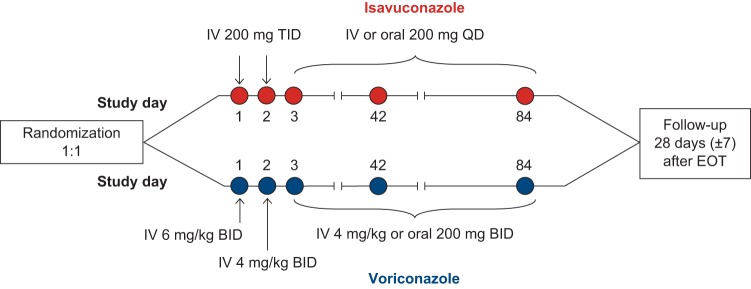
Study design. BID, twice daily; QD, once daily; TID, three times daily. Maximum therapy duration was 84 days.

The morbidity and mortality from invasive fungal diseases remain substantial (1). Triazole antifungal agents are first-line agents for the prevention and treatment of these infections. Voriconazole is recommended as primary treatment for invasive aspergillosis (IA). Posaconazole is primarily indicated as salvage therapy for patients with IA and prophylaxis for patients with neutropenia and hematopoietic stem cell transplant recipients (2). Isavuconazole administered as the prodrug isavuconazonium sulfate is a novel broad-spectrum triazole antifungal agent. Isavuconazonium sulfate has been approved by the U.S. Food and Drug Administration for the treatment of adults with IA and invasive mucormycosis (3) and by the European Medicines Agency for the treatment of adults with IA and those with mucormycosis for whom amphotericin B is not appropriate (4). In the SECURE clinical trial (ClinicalTrials.gov registration no. NCT00412893) (Fig. 1), isavuconazole was demonstrated to be noninferior to voriconazole for the primary treatment of invasive mold disease caused by Aspergillus and other filamentous fungi, as determined using all-cause mortality through day 42 as the primary endpoint (19% versus 20%, respectively) (5). Overall response and clinical response rates were similar for isavuconazole and voriconazole (50% versus 47% and 62% versus 60%, respectively), and the isavuconazole group had significantly lower rates of hepatobiliary disorders (9% versus 16%), eye disorders (15% versus 27%), skin or subcutaneous tissue disorders (33% versus 42%), and drug-related adverse events (42% versus 60%).

A deep understanding of the relationship between drug exposure and response is required to establish clinically useful threshold values for drug exposure for both clinical outcomes and adverse events. Exposure-response relationships for efficacy are well established for other currently approved triazoles, such as itraconazole, posaconazole, and voriconazole, which has led to target drug concentrations that are necessary to maintain drug levels within safe and effective ranges (6–10). Exposure-response relationships for safety are also well established for itraconazole and voriconazole (8, 11). Thus, an important question remains as to whether these relationships are also evident for isavuconazole. Establishing clinically relevant exposure-response and exposure-safety relationships will inform guidelines with respect to the potential need for therapeutic drug monitoring (TDM).

In the SECURE trial, plasma isavuconazole concentrations were available for the majority of patients who were enrolled in the isavuconazole arm. Therefore, this *post hoc* analysis was conducted to evaluate the exposure-response relationships in terms of efficacy and safety for isavuconazole using those patient data. Logistic regression modeling was used to explore the potential relationships between various measures of isavuconazole exposure and both clinical outcomes and adverse events.

## RESULTS

### Data for analysis.

Two hundred thirty-one patients from a previously developed population pharmacokinetic (PPK) model provided exposure parameters (12) used in the exposure-response analysis for both clinical outcomes and safety. One hundred twenty-nine patients qualified for the modified intent-to-treat (mITT) population based on data review committee (DRC)-adjudicated criteria. A summary of the covariates used in the analysis is provided in Table 1.

**TABLE 1:** Summary of patient characteristics^*a*^

Patient characteristic	ITT population^*b*^ (*n* = 231)		mITT population (*n* = 129)	
	Yes (*n*)	No (*n*)	Yes (*n*)	No (*n*)
Hematological malignancy	191	40	100	29
Uncontrolled malignancy	156	75	79	50
Neutropenia	150	81	79	50
Elevated serum galactomannan at baseline^*c*^	54	150	51	62
Lower respiratory tract disease	182	49	104	25

a Median duration of therapy for the ITT population, 51 days, and for the mITT population, 59 days.

b Yes/no, had/did not have characteristics at baseline. *n* is the number of patients.

c There was no galactomannan information for some patients (*n* = 27) at baseline.

### Exposure-efficacy analysis.

The exposure parameters are summarized in Table 2. The mean calculated exposure at steady state (total area under the concentration-time curve at steady state [AUCss]) was 101 mg · h/liter, with exposures ranging from 10 to 343 mg · h/liter. Mean trough concentrations at steady state (Css), trough concentrations after 7 days of dosing (C7), and trough concentrations after 14 days of dosing (C14) were approximately 3,600 ng/ml, 2,600 ng/ml, and 3,000 ng/ml, respectively. Trough concentrations ranged from 174 to 10,000 ng/ml.

**TABLE 2:** Summary of exposure parameters

Parameter	Value^*a*^		
	Mean (SD)	Median	Range
AUCss (mg · h/liter)	101 (56)	90	10–343
Css (ng/ml)	3,633 (2,023)	3,218	174–10,969
C7 (ng/ml)	2,631 (1,033)	2,477	189–5,627
C14 (ng/ml)	3,049 (1,397)	2,923	174–7,512

a Values are rounded to the nearest whole number. AUCss, total area under the curve at steady state; Css, concentration at steady state; C7, concentration after 7 days of dosing; C14, concentration after 14 days of dosing; SD, standard deviation.

### All-cause mortality at day 42.

**FIG 2:**
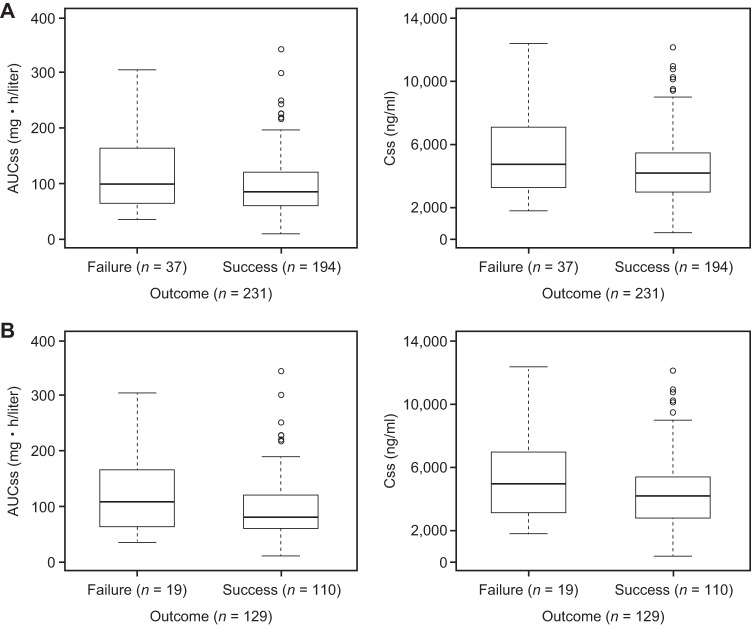
Box-and-whisker plots of drug exposure (AUCss and Css) versus mortality at day 42 for the ITT population (A) and the mITT population (B). Boxes represent the median and 25th and 75th percentiles, whiskers represent the range of maximum and minimum values within 1.5× the interquartile range, and outliers are shown as circles.

All drug exposure parameters (i.e., AUCss, Css, C7, and C14) were examined graphically and were modeled univariately. There was no apparent relationship between drug exposure parameters and mortality at day 42 for either the intent-to-treat (ITT) population or the mITT population (Fig. 2A and B, respectively). None of the primary parameters were retained in the logistic regression model. Logistic regression analysis did not suggest any positive association between exposure parameters and mortality at day 42. Since none of the primary exposure parameters were retained in the model, further covariate analysis was not performed.

### DRC-adjudicated overall and clinical responses at end of treatment (EOT).

**FIG 3:**
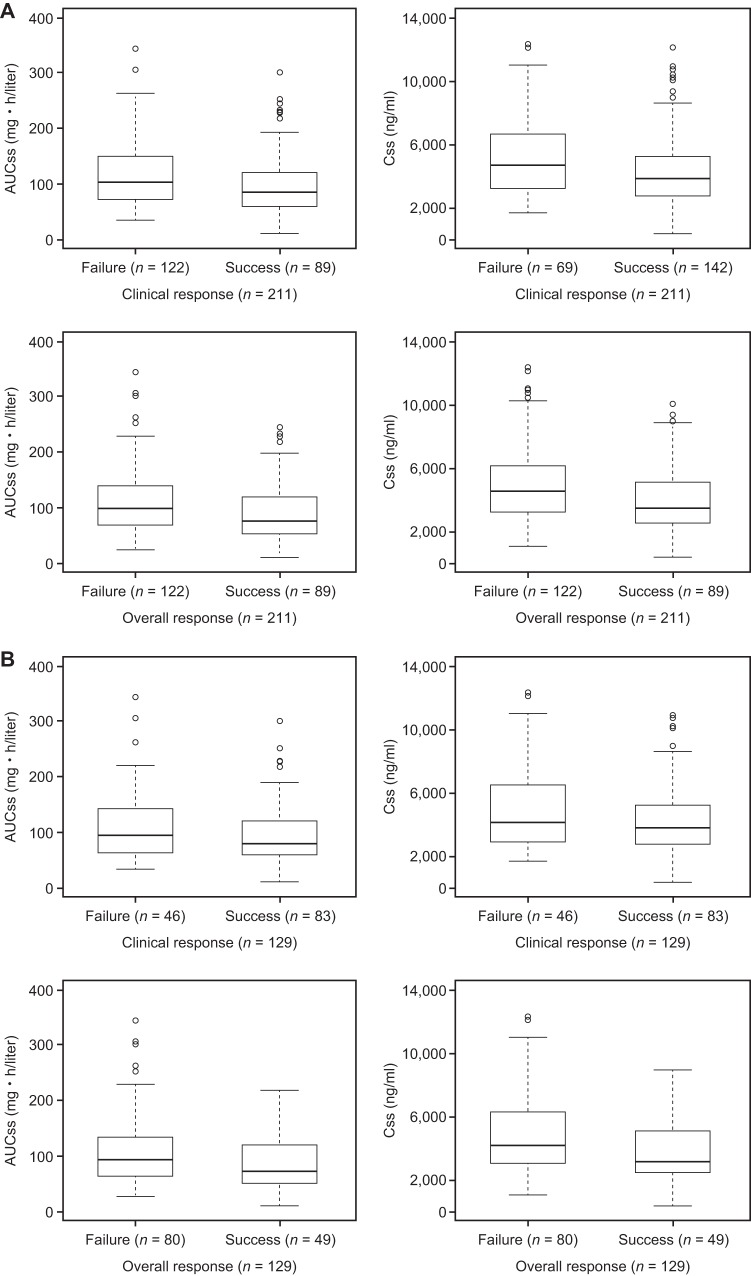
Box-and-whisker plots of drug exposure (AUCss and Css) versus clinical and overall responses at EOT for the ITT population (A) and the mITT population (B). Boxes represent the median and 25th and 75th percentiles, whiskers represent the range of maximum and minimum values within 1.5× the interquartile range, and outliers are shown as circles.

Graphical examination of binary outcomes for AUCss and Css for the ITT and mITT populations against clinical and overall responses are shown in Fig. 3A and B, respectively. Logistic regression models did not demonstrate any relationship of drug exposure with mortality, clinical response, and overall response. None of the exposure parameters were statistically significant (at a significance level of 0.05) and were not in the model. Similar results were obtained for C7 and C14 (data not shown).

### AUC/MIC calculations.

There was only a small sample subset of patients with both pharmacokinetic (PK) parameters and pathogen susceptibility data available (*n* = 36) compared with the total number of subjects in the study. Details of patients with MIC values are provided in Table S1 in the supplemental material. No significant relationship (*P* > 0.05) was identified between the AUC/MIC ratio and mortality at day 42, the overall response at EOT, or the clinical response at EOT. Since only 2 of the 36 patients were not included in the mITT population, the analysis would necessarily have yielded almost identical results, so it was not performed. No relationship was observed between MIC values and outcome parameters (13).

### Exposure-safety analysis.

**FIG 4:**
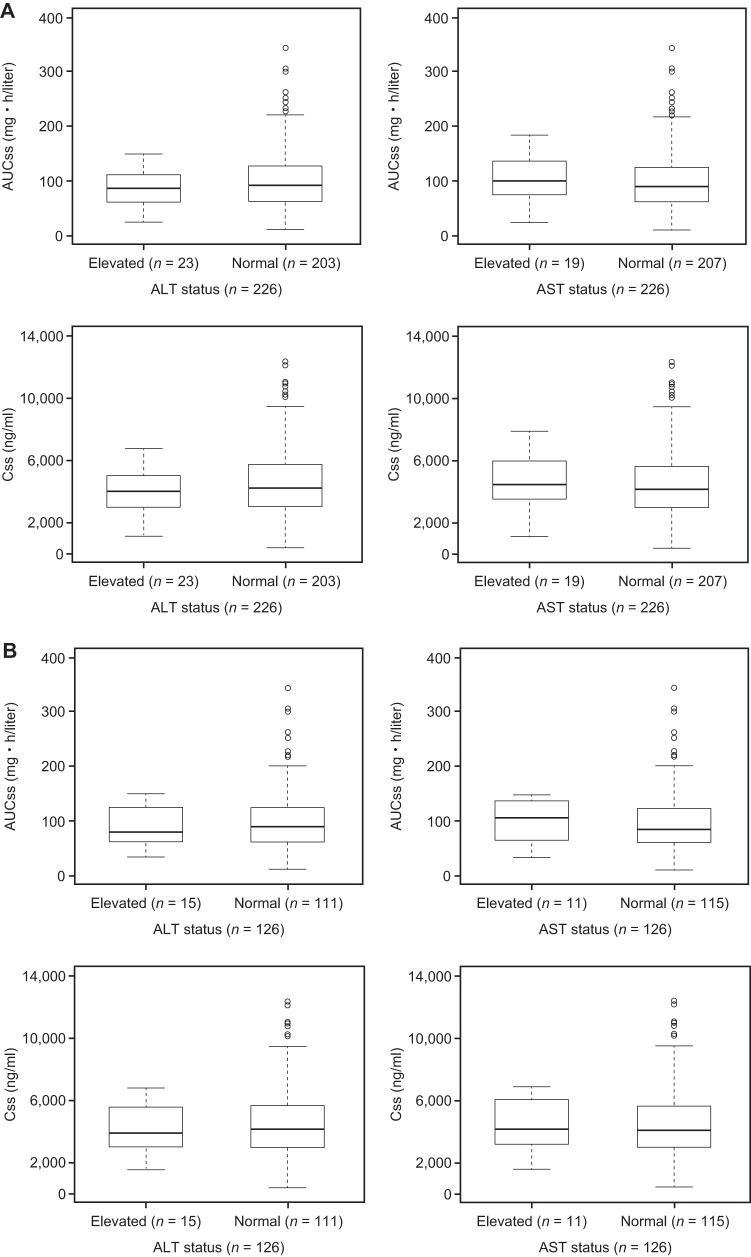
Box-and-whisker plots of drug exposure (AUCss and Css) versus ALT/AST levels at EOT for the ITT population (A) and the mITT population (B). Boxes represent the median and 25th and 75th percentiles, whiskers represent the range of maximum and minimum values within 1.5× the interquartile range, and outliers are shown as circles.

Patients with PK parameters used in the exposure-response analysis were also included in this analysis. Graphical examination of binary outcomes for AUCss and Css for the ITT and mITT populations against normal/elevated levels of alanine aminotransferase (ALT) and aspartate aminotransferase (AST) are shown in Fig. 4. None of the primary exposure parameters were found to be statistically significant for any of the safety outcomes (ALT or AST or combined ALT and AST) for either the ITT (*n* = 226) or mITT (*n* = 126) population. As none of the primary exposure parameters were significant (*P* > 0.3), there was no retention of parameters in the logistic model.

## DISCUSSION

The primary aim of this analysis was to investigate any potential relationship between various measures of drug exposure of isavuconazole and both efficacy and safety outcomes. Such an understanding is required to further reflect on the potential requirement for TDM as a component of routine clinical care of patients receiving isavuconazole. Conducting an exposure-response/safety analysis provides an understanding of any threshold of exposure that is predictive of efficacy and/or adverse events.

We were unable to demonstrate any statistically significant relationships for any measure of drug exposure (i.e., AUCss, Css, or AUC/MIC) and various outcomes (i.e., all-cause mortality at day 42 or clinical and overall responses at EOT or MIC of fungal isolates). A slight trend was observed for overall responses for both the ITT and mITT populations, but it was not statistically significant (*P* > 0.05).

There could be several reasons for the lack of any relationship between drug exposure and clinical outcomes from this analysis. First, even though there were some extremes in predicted exposures, the variability was only 62% in the patient population (12). Second, it is possible there was a degree of bias in the PPK model. The PPK model was fitted to data from phase 1 and sparse data from phase 3. Even though there were 231 patients in the SECURE study, the sparse data may potentially have led to biased estimates of exposure and Css values. However, there is no evidence of this, given concordance with PK models fitted to other isavuconazole data sets (14). Poor compliance with the study drug could also have led to biased estimates of drug exposure, although there is no specific evidence to suggest this occurred. Alternatively, assuming the existence of a sigmoidal exposure-response relationship, the lack of a relationship with outcomes might simply reflect the fact that exposures were on the plateau of the curve (suprathreshold). The lack of association between exposure and response is consistent with the proposition that the isavuconazole exposures achieved by the clinical dosage regimen were near maximal for treating the infecting organisms in the SECURE study. In this respect, it is worth noting that the overall cure rate observed for isavuconazole in the SECURE trial was comparable to those in other trials of triazole antifungals (2, 5, 15, 16).

Although isolates were not obtained from the majority of patients (and therefore MIC values for the invading pathogens were not determined), it is likely that most patients were infected by wild-type organisms. It is possible that the inclusion of more patients infected with non-wild-type strains might have enabled exposure-response relationships to be better described. *In vivo* and *ex vivo* models have demonstrated that the MIC values have a clear impact on exposure-response relationships, as proportionally higher drug exposures are required to achieve the same outcomes for strains with higher MICs (17–21). Although there were insufficient numbers of patients in the SECURE study for whom pathogen susceptibility was the only distinction to allow that possibility to be tested, a few patients with MIC values of up to 8 mg/liter were successfully treated (5). However, ongoing information from the postlicense database may eventually enable clinical exposure-response relationships to be better defined.

Even though a threshold value for any drug exposure parameter was not found to be correlated with mortality and clinical response, the duration of therapy did appear to be important and was statistically significant (*P* < 0.05). This finding should be interpreted with some caution. The importance of the duration of therapy may be confounded by other factors that influence outcomes (e.g., the nature of the underlying disease). There is currently no definitive evidence that suggests that longer duration of therapy is necessarily associated with a better clinical response. Furthermore, there is no clear clinical evidence of the minimum duration of antifungal therapy that is required for clinical cure.

Hepatotoxicity is a class effect for the azole group of antifungal agents, with effects ranging from mild increase in liver function tests to possibly fatal hepatic failure being reported (22). The exact mechanism of elevated liver function tests with azole antifungal agents remains unknown (22). Due to the primary concern with elevated liver function test values, exposure-safety analysis was performed on elevated ALT and AST levels. These values were available for all the patients. The current analysis did not identify any association between isavuconazole exposure and elevated ALT or AST levels or for a combination of both ALT and AST levels. One limitation of this analysis is the small proportion of patients who had elevated ALT or AST levels. Only 23/226 and 19/226 patients in this analysis had elevated ALT or AST levels, respectively.

Voriconazole, posaconazole, and itraconazole have target trough concentrations that need to be maintained in order optimize the probability of response. The voriconazole target recommended by the British Society of Medical Mycology is between 1.0 and 5.5 mg/liter when the drug is used to treat invasive infection (7). The target voriconazole concentrations for prophylaxis are less clear. For posaconazole, the target trough concentrations are >0.7 μg/ml for prophylaxis and >1 mg/liter for salvage therapy. For itraconazole, the target trough concentrations are similar to those for voriconazole (7). Fluconazole does not require routine therapeutic drug monitoring. There is no apparent relationship between exposure and efficacy to suggest routine TDM for isavuconazole. However, it is reasonable to continue observing real-world patients who are administered isavuconazole and to monitor their exposures when necessary to ensure they do not require TDM. There might be a necessity to confirm isavuconazole exposures in select clinical cases (e.g., severe gut disease from graft-versus-host disease [in which drug absorption through the oral route is problematic], in treatment of central nervous system infections, or in infections with non-wild-type fungal pathogens). TDM may also be necessary when dosing in children or adolescents due to minimum exposure information (23).

In conclusion, no statistically significant relationships were observed for any of the exposure parameters of isavuconazole (AUCss, Css, C7, and C14) with any safety markers (ALT, AST, and combined ALT and AST), either at the EOT or postbaseline, or with any efficacy endpoints (all-cause mortality and overall and clinical responses). In some models, duration of therapy was retained in the model. However, this covariate is highly confounded, making its relevance in the analysis unclear. Also, experimental pharmacodynamic models were conducted to establish the exposure-response relationship associated with efficacy and to estimate the target exposure associated with the optimal exposure-response relationship. The results showed that the clinical dosing regimen achieved exposures adequate to treat infections. All the models were developed based on the observed data (12); however, the models were not validated against external data from a clinical trial, which would have required performing additional isavuconazole studies.

Finally, TDM may be considered for individual cases, as discussed above, but at present, there is no clear evidence that there is a general need for TDM or a clear target to recommend.

## MATERIALS AND METHODS

### Study design.

SECURE (ClinicalTrials.gov registration no. NCT00412893) was a global, phase 3, randomized, multicenter, double-blind, parallel-group noninferiority trial (Fig. 1). Full details of the SECURE trial have been published previously (5).

Patients with proven/probable disease, as assessed by an independent and blinded DRC, were included in the mITT population. All the patients received 372 mg of isavuconazonium sulfate (equivalent to 200 mg isavuconazole) administered by intravenous (i.v.) infusion every 8 h for 6 doses (i.e., days 1 and 2), followed by a maintenance dose of 372 mg isavuconazonium sulfate administered once daily, either i.v. or orally (p.o.), from day 3 to EOT. Here, only isavuconazole and the dosing equivalent were used.

### Efficacy and safety assessments.

In the current analysis, the efficacy endpoints included were (i) all-cause mortality through day 42 in the ITT and mITT populations, (ii) DRC-adjudicated overall response at EOT in the ITT and mITT populations, and (iii) DRC-adjudicated clinical response at EOT in the ITT and mITT populations. Liver function test values (AST and ALT) at the EOT and postbaseline (EOT plus 10 days) were assessed as safety outcomes.

### Estimation of pharmacokinetic (exposure) parameters.

A PPK model was previously developed for concentration data from the SECURE study in combination with data from healthy subjects, using NONMEM version 7.2 (GloboMax LLC, Hanover, MD, USA) (12). This publication lists values and dispersions associated with parameters that were used for the simulation. The AUCss was calculated using the standard formula (AUC = *F* × dose/CL, where *F* is bioavailability and CL is clearance) based on the individual parameter estimates from the best PPK model. Individual parameter estimates obtained from the best model with covariates were used to calculate Css, C7, and C14.

### Exposure-response analysis.

All the efficacy and safety data were evaluated as binary and ordinal data using a logistic regression model in SAS (version 9.3; SAS Institute Inc., Cary, NC, USA). The graphic processing of the data was also performed in SAS or R (version 2.17; available at https://www.r-project.org) (24). Each efficacy endpoint and safety endpoint described above was analyzed separately using isavuconazole exposure parameters.

The covariates were identified based on scientific interest or prior knowledge of any possible relationship with exposure parameters. Duration of therapy was the only continuous covariate investigated. The categorical covariates tested for the exposure-efficacy analysis included race (Caucasian/Asian), hematological malignancy (yes/no), uncontrolled malignancy at baseline (yes/no), neutropenia at baseline (yes/no), serum galactomannan at baseline (<1/≥1), and lower respiratory tract disease (yes/no). Covariates, along with primary exposure parameters, were added in an automated stepwise approach (α = 0.3 for model inclusion and α = 0.05 for model retention).

Exposure-response analyses were also performed for patients in the ITT population who had MIC values for any Aspergillus spp. (including A. flavus, A. fumigatus, A. niger, and A. terreus). MIC values were determined by Case Western Reserve University, Cleveland, OH, USA, using the European Committee on Antimicrobial Susceptibility Testing (EUCAST) methodology (25). AUC_0-∞_/MIC ratios were calculated based on model-predicted AUCss values for a patient and the corresponding highest MIC value, irrespective of the fungus that was cultured.

## Supplementary Material

Supplemental material
