# Supplementary material for: Exposure-Response Relationships for Isavuconazole in Patients with Invasive Aspergillosis and Other Filamentous Fungi
Source: Antimicrob Agents Chemother. 2017 Nov 22;61(12):e01034-17. doi: 10.1128/AAC.01034-17 (PMC5700339; doi:10.1128/AAC.01034-17)
Supplement: Supplemental material [file supp_61_12_e01034-17__index.html]

Supplemental material 

# Exposure-Response Relationships for Isavuconazole in Patients with Invasive Aspergillosis and Other Filamentous Fungi

## Supplemental material

- Supplemental file 1 -

  Table S1

  PDF, 17K
