# Supplementary material for: Exposure-Response Relationships for Isavuconazole in Patients with Invasive Aspergillosis and Other Filamentous Fungi
Source: Antimicrob Agents Chemother. 2017 Nov 22;61(12):e01034-17. doi: 10.1128/AAC.01034-17 (PMC5700339; doi:10.1128/AAC.01034-17)
Supplement: Supplemental material [file AAC.01034-17_zac012176699s1.pdf]

Supplementary Table S1 Characteristics and outcomes for patients having minimum inhibitory concentrations for pathogens

	A. fumigatus	*A. flavus*	*A. terreus*	*A. niger*	Mixed *Aspergillus* spp.	Mixed *Aspergillus* spp. + other spp.
Total	17	6	2	5	4	2
Proven / Probable / No IFD confirmed, N	6 / 10 / 1	2 / 3 / 1	1 / 1/ 0	1 / 4/ 0	1 / 3/ 0	
Median Treatment Duration, days (range)	83 (13-91)	84 (49-88)	77 (56-98)	52 (11-84)	82 (35-84)	25.5 (6-45)
CLSI MIC range, mg/L	0.25 – 4	1-4	1-4	2-4	0.25-2	0.5-16
ACM D42, n (%)	1 (6)	0	0	1 (20)	0	1 (50)
ACM D84, n (%)	2 (12)	0	0	2 (40)	1 (25)	1 (50)
Successful OR D42, n (%)	5 (29)	3 (50)	1 (50)	4 (80)	2 (50)	1 (50)
Successful OR EOT, n (%)	6 (35)	3 (50)	0	3 (60)	2 (50)	1 (50)
Median AUCss, ng*h/mL (range)	59,799 (33,043-229,189)	91,100 (55,237-186,046)	88,199 (68,704-107,694)	110,326 (59,806-138,054)	61,512 (49,185-134,354)	55,029 (50,082-59,975)

ACM, all-cause mortality; AUCss, area under the time-concentration curve at steady state; CLSI, Clinical & Laboratory Standards Institute; D42, Day 42; D84, Day 84; EOT, end of treatment; IFD, invasive fungal disease; MIC, minimum inhibitory concentration; OR, overall response
